# The rise of India’s global health diplomacy amid COVID-19 pandemic

**DOI:** 10.34172/hpp.2023.34

**Published:** 2023-12-16

**Authors:** Vijay Kumar Chattu, Bawa Singh, Fnu Kajal, Chakrapani Chatla, Soosanna Kumary Chattu, Sanjay Pattanshetty, K. Srikanth Reddy

**Affiliations:** ^1^Department of Occupational Science & Occupational Therapy, Temerty Faculty of Medicine, University of Toronto, Toronto, ON M5G 1V7, Canada; ^2^Center for Global Health Research, Saveetha Medical College and Hospitals, Saveetha Institute of Medical and Technical Sciences, Saveetha University, Chennai 600077, India; ^3^Department of Community Medicine, Faculty of Medicine, Datta Meghe Institute of Medical Sciences, Wardha-442107, India; ^4^Department of South and Central Asian Studies, School of International Studies, Central University of Punjab, Bathinda 151401, India; ^5^Department of Health Promotion Sciences, University of Arizona, Tucson, AZ 85719, USA; ^6^Global Health Equity, Public Health Department, Thermo Fisher Scientific, Hyderabad, Telangana; ^7^Center for Evidence-based Diplomacy (CEBD), Global Health Research and Innovations Canada (GHRIC), Toronto, ON, Canada; ^8^Department of Global Health Governance, Prasanna School of Public Health, Manipal Academy of Higher Education, Manipal, India; ^9^School of Epidemiology and Public Health, University of Ottawa, Ottawa

**Keywords:** Global health, Health diplomacy, India, Policy, COVID-19, Pandemic, Vaccines, Pharmaceuticals, Essential medicines

## Abstract

The COVID-19 pandemic has highlighted the importance of global health diplomacy (GHD), with India emerging as a key player. India’s commitment to GHD is demonstrated by its active participation in regional and multilateral projects, pharmaceutical expertise, and large-scale manufacturing capabilities, which include the production and distribution of COVID-19 vaccines and essential medicines. India has supported nations in need through bilateral and multilateral platforms, providing vaccines to countries experiencing shortages and offering technical assistance and capacity-building programs to improve healthcare infrastructure and response capabilities. India’s unique approach to GHD, rooted in humanitarian diplomacy, emphasized collaboration and empathy and stressed the well-being of humanity by embracing the philosophy of "Vasudhaiva Kutumbakam," which translates to "*the world is one family*." Against this background, this paper’s main focus is to analyze the rise of India’s GHD amidst the COVID-19 pandemic and its leadership in addressing various global challenges. India has demonstrated its commitment to global solidarity by offering medical supplies, equipment, and expertise to more than 100 countries. India’s rising global leadership can be attributed to its proactive approach, humanitarian diplomacy, and significant contributions to global health initiatives.

## Introduction

 The COVID-19 pandemic has highlighted the importance of global health diplomacy (GHD) in addressing global health challenges. India, a prominent player in this diplomatic endeavor, has embraced its ancient philosophy of “Vasudhaiva Kutumbakam,” which translates to “*the world is one family*”.^1^ India’s ascent in GHD has been catalyzed by its strong pharmaceutical industry and vast manufacturing capabilities, making it a key supplier of essential medicines and vaccines, producing 60% of the world’s vaccine supply.^2^ India actively engages in health diplomacy at both regional and multilateral levels, recognizing the importance of collective actions and collaborations. Through platforms such as the World Health Organization (WHO), the South Asian Association for Regional Cooperation (SAARC), and other bilateral engagements, India has fostered healthcare collaboration and partnerships, shared expertise, and coordinated responses to mitigate the pandemic’s impact and strengthen global health systems.

 India’s approach to GHD is characterized by a humanitarian perspective, emphasizing empathy and compassion in addressing health crises. This approach prioritizes the well-being of humanity and calls for a collective response that transcends political boundaries. India’s humanitarian approach ensures equitable access to healthcare, bolsters healthcare infrastructure, and fosters cooperation for the greater benefit of all nations. To examine and analyze the objective of this paper, the authors raised four questions to reach the logical conclusion of this perspective: what is GHD? What were the disparities and inequalities in vaccine distribution during the pandemic? What were the factors contributing to India’s capacity for vaccine production? and how India has responded to these vaccine inequalities and disparities?

## Global health diplomacy

 GHD has become an essential strategy to encourage and enhance international coordination and cooperation to address urgent health issues/challenges/concerns, as evident from the international community to successfully respond to epidemics, pandemics, and other health emergencies by utilizing diplomatic tools like negotiations, alliances, and information sharing.^3^ Governments can emphasize health through GHD, realizing that it is an essential component of their foreign policy agenda and that a healthy populace helps overall stability and prosperity. By providing technical expertise, coordinating responses, and advocating for fair access to healthcare resources, international/regional organizations like the WHO, the European Union (EU), the Association of Southeast Asian Nations (ASEAN), and SAARC have played a crucial role in supporting GHD activities. Non-state actors, such as charitable foundations and civil society groups, make important contributions by mobilizing funds, increasing public awareness, and promoting legislative reforms to address global health issues. These numerous stakeholders collaborate to develop laws and agreements that support universal healthcare, improve healthcare delivery, and address social determinants of health. GHD opens the door for creative solutions by encouraging cross-sector and international collaboration.

 GHD is a relatively new discipline; scholars have discussed and contested it from various perspectives. According to Kickbusch et al, GHD is a multi-level, multi-actor negotiation mechanism that shapes and influences the global healthcare environment.^4^ Labonte and Gagnon have stressed that global health has become one of the most important foreign policy issues at the current time, and this new approach to global health is called “Global Health Diplomacy,” which includes the process and the involvement of all key stakeholders.^5^ According to Fauci health diplomacy is “winning the hearts and minds of people in poor countries by exporting medical care, expertise, and personnel to help those who need it most”.^6^ Adams et al. argued, “successful health development efforts depend on functional and respectful relationships between all the stakeholders, including donor and recipient governments, health care providers, local political leaders, and field-based NGOs.”^7^ For health diplomats to succeed, whether working in a clinic or at the policymaking table, they must have a deep understanding of these relationships’ structures, programs, approaches, and pitfalls. According to Chattu and Knight, health diplomacy in the modern world is a “political activity with the dual goals of improving health and keeping and strengthening international relations abroad.”^8^

 Examining how global health issues are incorporated into foreign policy agendas and how foreign policy tools are used to address global health challenges is one way to understand the connections between foreign policy and GHD. Moreover, health is gaining an important space and significant attention in foreign policy due to its increasing importance in three major global agendas. Health security is propelled by the anticipated pandemic apprehension or the deliberate dissemination of pathogens and the escalation of humanitarian conflicts, natural calamities, and unforeseen crises. The economic perspective is another issue to consider health as an important part of foreign policy. The economic perspective considers the negative impacts of poor health on development or the consequences of pandemic outbreaks on the global market and the potential benefits derived from the expanding global market for health-related goods and services. The concept of social justice encompasses reinforcing health as a fundamental social value and a basic human right. It involves actively supporting the United Nations Sustainable Development Goals (SDGs), advocating for equitable access to essential medicines and primary healthcare services, and urging HICs to allocate substantial resources towards diverse global health initiatives.^9^ Some countries may seek to increase their “soft power,” “promote national interests,” and “foster cooperation and trust” through GHD. Conversely, some countries may have to prioritize other foreign policy priorities over their global health obligations.^10^

 COVID-19 recently highlighted the importance of healthcare as a foreign policy issue for large countries. Health diplomacy is being used by several countries as a form of soft power to further their national and international goals. There has been a rise in awareness of the importance of improving healthcare across the globe. It is a synthesis of population-based prevention and individual-level clinical care. The significance of GHD has grown exponentially over the years as healthcare has become a transnational issue requiring more effective collaborative actions. Both global healthcare and international relations benefit from the GHD. According to Chattu, GHD has gained traction since numerous players outside the WHO debate healthcare issues to create global policy for distinct health determinants.^11^

 The WHO has identified three main goals of health diplomacy: (*a*) to improve health security and population health; (*b*) to improve interstate relations and a commitment by various actors to improve health; and (*c*) to the achievement of outcomes that are regarded fair and promote the goals of reducing poverty and increasing equity.^12^ In their paper, Mol et al pointed out that “health diplomacy can be useful for disease identification, prevention, and responding to health issues, and through GHD, medical assistance and humanitarian aid are provided during an emergency.” Health diplomacy provides reciprocal benefits and binds societies by allowing cooperating countries to leverage soft power to promote multidimensional interests.^13^

## COVID-19 and vaccine disparities and inequities

 COVID-19 has exposed the world’s binary regarding vaccine disparity and inequity.^14^ Disparity and inequity are related but distinct concepts that enlighten ethical and moral discourses in medical practice and health policy. Inequity denotes unfairness and injustice, whereas disparity implies some form of difference. U.S. Department of Health & Human Services defines health disparities as “differences that occur by gender, race or ethnicity, education or income, disability, geographic location, or sexual orientation”^15^. Similarly, LaVeist and Isaac defined inequity as “differences in the incidence, prevalence, mortality, and burden of diseases and other adverse health conditions that exist among specific population groups in the United States.”^16^ Linguists such as Chomsky hold the belief that thought and mental processes become skewed when essential terms are severed from their natural context and given a specialized meaning.^17^ For example, when a value-free term (such as disparity) is repeatedly associated with a value-laden term (such as inequity), the value-free term eventually becomes value-laden, which obscures its actual meaning and application.

 Another example would be when a value-laden term is repeatedly associated with a value-free term (such as inequality). However, it is concerning that the science of healthcare disparities suffers from a conceptual lag due to how healthcare disparities are studied and interpreted.^18^ The term disparity is frequently associated with discrimination in the prevailing literature. This prevents providers and the general public from looking beyond the expression to the sources of disparities, and it impedes appropriate measurement and application of this extremely complex concept.

 Regarding socioeconomic and geopolitical health determinants, vaccine inequality and equity have resulted in several political, economic, social, and international factors. Concomitantly, COVID-19 has also seriously impacted and resulted in significant vaccine disparities and inequity between developed and developing countries. Given the resources, science & technology, and capital, the world has been divided into two groups regarding access to medicines and vaccines: the haves and the have-nots.^19^ Conversely, wealthy nations reacted with narrow self-interest, hoarding the world’s then-limited supply for themselves.^20^ While a significant portion of the population in developed countries has received vaccinations, developing nations have had challenges in providing vaccines to their citizens. The COVID-19 pandemic is an outlier and equitable global access to the vaccine is required to contain the outbreak. The world, on the other hand, is witnessing the polar opposite, namely, vaccine nationalism. Vaccine nationalism is the “my country first” approach to acquiring and stockpiling vaccines before making them available to other countries.^21^

 A systematic review by Bayati et al highlighted disparities in global vaccine access. In December 2020, emergency regulatory approval was granted for the initial COVID-19 vaccines.^22^ In the initial year of vaccine distribution, high-income countries (HICs) have successfully achieved their vaccination goals by immunizing approximately 75%-80% of their respective populations. On the other hand, the same authors found a contrasting picture of the low-income countries (LICs) in this respect. These countries have been struggling to ensure equitable access to COVID-19 vaccines. Despite the urgent need for widespread vaccination, these LICs have encountered significant obstacles in their vaccination efforts, resulting in a low vaccination rate.^22^

 Another study by Tatar et al revealed a significant disparity in the distribution of COVID-19 vaccines. Their finding highlights the presence of a pronounced inequality in the allocation of vaccines.^23^ The distribution of COVID-19 vaccines has become a critical global concern due to the emergence of severe inequality in the distribution process. The analysis revealed a significant disparity, with approximately 80% of the global population having access to a mere 5% of the total COVID-19 vaccine supply. Conversely, the remaining 20% of the population has been allocated 95% of the available vaccines.^23^ This stark contrast highlights the unequal distribution of vaccines and underscores the urgent need for a more equitable approach to ensure widespread immunization coverage. Such disparities can have profound implications for public health and social well-being. Unequal access to vaccines can exacerbate the virus’s spread and prolong the pandemic’s duration. Moreover, wealth inequality can contribute to social unrest and hinder economic development.

**Figure 1:**
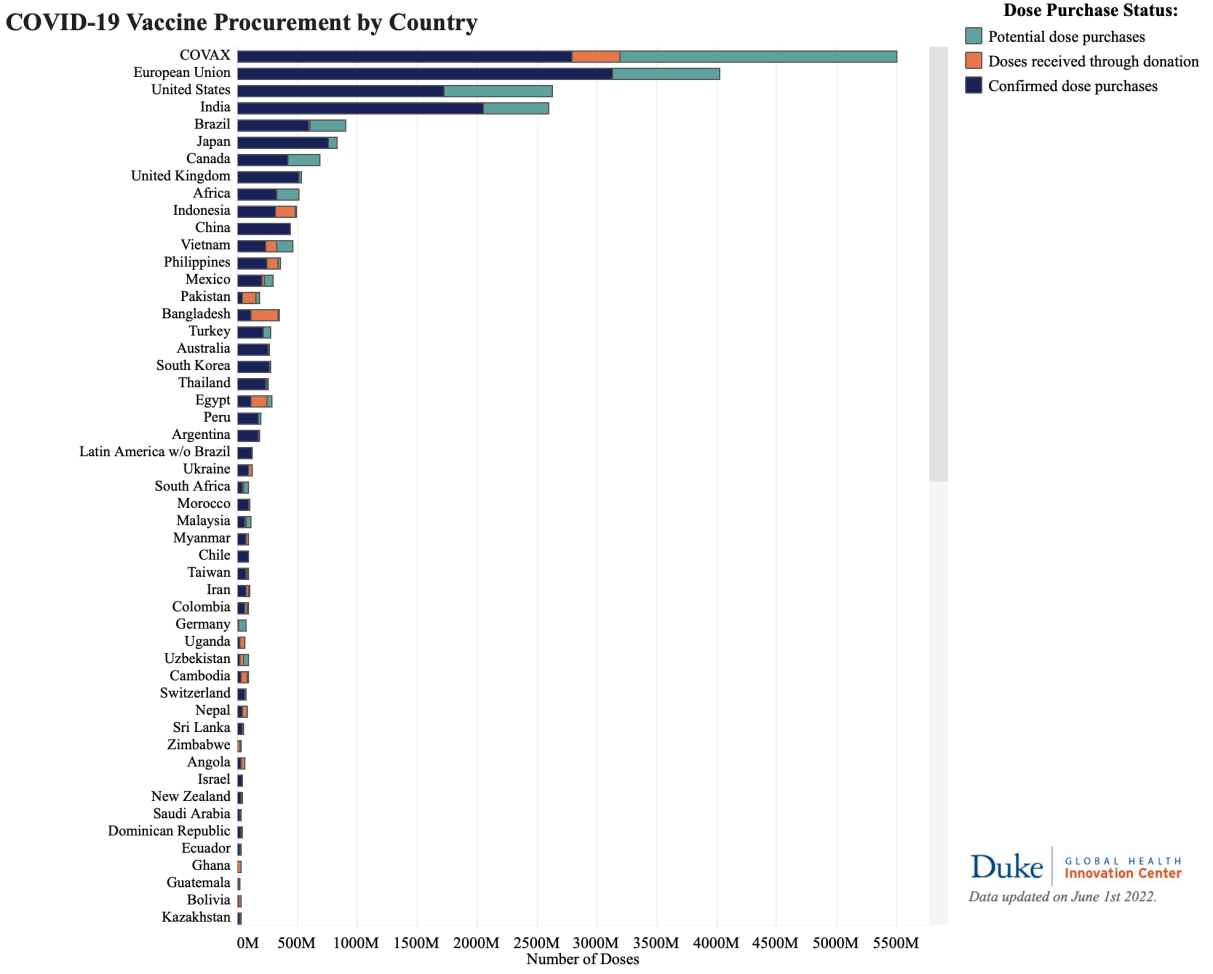
Procurement by high-income and the low-income countries. COVID-19 vaccine procurement by country. Source: https://launchandscalefaster.org/covid-19/vaccinepurchases. Accessed July 23, 2023

 Another study by Mathieu et al highlighted the stark contrast. The ongoing disparity in the distribution of vaccines stands as a prominent example of the significant shortcomings in international collaboration witnessed throughout the COVID-19 pandemic.^24^ In contrast to HICs, which have demonstrated swift progress in vaccinating a significant portion of their populations, other regions of the globe still face challenges in obtaining adequate supplies of COVID-19 vaccines.^25^ European and North American HICs have stockpiled far more vaccine doses than required (Duke University). The WHO Director-General reported that by November 2021, more than 80% of the world’s vaccines had been distributed to G20 countries, while LICs had received only 0.6% of all vaccines.^26^Figure 1 shows the disparities and inequities between the HICs and LICs.

 As of June 2022, the number of confirmed purchases amounts to 11.9 billion doses, while an additional 6 billion doses are being negotiated or reserved as optional expansions to current agreements.^27^ As of the 4th of July 2021, HICs have purchased more than half of all vaccine doses made available for purchase worldwide (6.16 billion). Only 0.3 percent of the vaccines produced during the same period were distributed to the low LICs. Low- and middle-income countries (LMICs), which account for 81% of the world’s adult population, purchased roughly 33% of the vaccine, with COVAX receiving the remaining 13%.^28^

## India rise through its GHD: a reliable and trustworthy partner

 The evidence mentioned above clearly shows that vaccine disparities and inequities have persisted during the peak of COVID-19 between HICs and LICs.^29^ Concomitantly, the pandemic has undoubtedly had a greater impact on poorer and developing countries. Moreover, the HICs have also not remained immune, exposing existing inequalities across countries.^30^ Due to their dearth of political will and solidarity, many developed nations retreated in the face of the pandemic. Under the Trump administration, even the United States had announced to withdraw from WHO at the pandemic’s peak.^31^ Moreover, in response to a medical equipment supply shortage, the US and Europe halted N95 mask exports and ordered a re-routing of their overseas production to meet domestic demand.^32^

 India’s response to the COVID-19 pandemic demonstrated its adherence to the doctrine of Vasudhaiva Kutumbakam. Despite the challenges posed by the situation, the country exhibited its dedication to world unity, solidarity, and humanitarianism.^33^ Here are a few examples of how India’s response to the epidemic, such as vaccine diplomacy, demonstrates its belief that everyone is part of one giant family. India has also made significant contributions to the fight against COVID-19 by joining regional and international conferences to coordinate efforts and share resources. India worked closely with the WHO and several other regional organizations, such as the SAARC, G-20, Quad (a diplomatic partnership between Australia, India, Japan, and the United States), African Union (AU), ASEAN, EU, etc. India has helped over 100 needy countries by sending them medical supplies, equipment, and experts. This shows how India is committed to supporting global healthcare systems and attempts to stop the spread of the virus by sharing medical expertise and knowledge, virtual training programs, etc. India has provided hydroxychloroquine to 55 countries, including four permanent UNSC members, namely the US, Russia, France, and the UK, its neighboring SAARC members, and 20 African countries. India showed its commitment and leadership as a member of the BRICS bloc (Brazil, Russia, India, China, and South Africa) by gifting medicines to 31 countries and exporting commercially to 24 other countries. India has provided pharma assistance to nearly 85 countries on a grant basis to help them tide over the COVID-19 pandemic.^34^

###  Global pharmacy depot 

 Secondly, by maintaining its potential as a “Global Pharmacy Depot” with its history of manufacturing and supplying many generic drugs at a very affordable price to developing countries, it has taken a proactive role in helping global citizens. According to a Turkish news broadcast, India’s pharmaceutical industry played a “crucial role” during the COVID-19 pandemic and cemented the country’s reputation as a “dependable nation” in health crises.^35^ Regarding the global market, India holds a significant share and is known as the world’s pharmacy and the largest generic supplier.^36^

 A joint report by Ernst & Young and Organizers of Pharmaceutical Producers of India (OPPI) highlighted that for decades, the Indian pharmaceutical industry has been recognized as the “pharmacy of the world.” The industry is widely recognized for its leadership in the global generics sector, accounting for over 20% of worldwide generics supply by volume and meeting nearly 60% of global vaccination demand.^37^ According to WHO, in only one year, the nation effectively distributed an impressive 1.56 billion vaccine doses, with 93% of the adult population obtaining the initial dose and 70% attaining complete vaccination. This accomplishment not only demonstrated one of the fastest vaccination campaigns in the world but also highlighted India’s expertise as the architect of the largest digital vaccination initiative in the world.^38^ The Indian pharmaceutical industry has an annual revenue of $38 billion (third largest in the world by volume and eleventh by value) and comprises 3000 pharma companies and 10 500 manufacturing facilities that produce drugs at around a third of the US costs and half of the European costs.^39^ India holds a prominent position in the international pharmaceutical industry, supplying 20% of the world’s generics and 62 percent of its vaccines.^39^ Over 80% of the antiretroviral medications used worldwide to combat AIDS are currently supplied by Indian pharmaceutical companies.^40^

 According to the predictions based on 2021 Indian Economic Survey, the domestic market will triple in size over the next decade. Domestic pharmaceutical sales in India totaled US$ 42 billion in 2021 and are projected to reach US$ 65 billion by 2024, and US$ 120-130 billion by 2030.^41^ India’s biotechnology industry includes biopharmaceuticals, bio-services, bio-agriculture, bio-industry, and bioinformatics. In 2020, the Indian biotechnology industry was valued at $ 70.2 billion; by 2025, it is projected to reach $150 billion.^42^ In FY20, the market for medical devices in India was valued at $10.36 billion. From 2020 to 2025, the market is anticipated to grow at a CAGR of 37% to reach $50 billion.^43^

 By volume of production, the Serum Institute of India (SII) is the largest vaccine manufacturer in the world.^44^ SII produces the COVID-19 vaccine known as Covishield, developed by Oxford-AstraZeneca and India’s most widely used COVID-19 vaccine.^45^ Covaxin, India’s first COVID-19 vaccine, was developed by Bharat Biotech in collaboration with the National Institute of Virology (NIV), part of the Indian Council of Medical Research (ICMR).^46^Additionally, Bharat Biotech is one of the first companies to develop vaccines for the Zika and Chikungunya viruses, making it a pioneer in this field.^47^ Zydus Lifesciences is responsible for developing the first human DNA COVID-19 vaccine and India’s second indigenous COVID-19 vaccine.^48^

 It is home to half a dozen major vaccine makers for polio, meningitis, pneumonia, rotavirus, BCG, measles, mumps, and rubella, among other diseases. Amid the COVID-19 pandemic, around half a dozen Indian firms, in collaboration with the US and UK, are developing vaccines against the virus, e.g., the SII, the world’s largest vaccine maker by the number of doses, makes 1.5 billion doses every year supplying around 20 vaccines to 165 countries is developing a “live-attenuated” vaccine in collaboration with American biotech. During this pandemic, it has also exported huge consignments of HCQ and paracetamol tablets to at least 100 countries. India has been in regular contact with all Gulf Cooperation Council (GCC) countries, and after several Gulf countries requested the supply of medicines like HCQ and paracetamol, India has fulfilled their requests. Having these pharmaceutical strengths and being a humanitarian practitioner, evident during the pandemic’s peak, India has risen to the occasion and justified its global leadership.

###  India’s health diplomacy at regional and multilateral levels

 Thirdly, India’s health diplomacy endeavors at regional and multilateral platforms during the COVID-19 pandemic have significantly contributed to managing the pandemic health emergency. India has demonstrated a proactive approach in engaging with more than 100 countries on a bilateral basis to extend medical aid and assistance. India has gained recognition as a prominent global supplier of COVID-19 vaccines, earning the name “pharmacy of the world.” The SII was crucial in producing the Oxford-AstraZeneca vaccine (Covishield) through a licensing agreement. India distributed vaccines not only to its neighboring countries in South Asia as well as various nations in Africa, Latin America, and the Caribbean as part of its Vaccine Maitri (Vaccine Friendship) program.^49^

 At the regional level, India made an initial contribution of US$10 million towards establishing the SAARC COVID-19 Emergency Fund to support initiatives to address the pandemic in the South Asian region. India convened a video conference of SAARC leaders to deliberate on collaborative strategies and facilitate the exchange of pertinent information about managing the ongoing pandemic. It has also launched several cooperation programs to combat Tuberculosis and HIV/AIDS and initiated the South Asia Vaccine Action Plan.^50^As highlighted by Mol et al, the list of various cooperative programs is quite exhaustive, such as G20, ASEAN, QUAD, AU, EU, etc.^13^

 India contributed to G20 pandemic preparedness response debates and advocated for equal access to immunizations, diagnostics, and treatments, especially for LMICs and under its leadership, admitted the African Union as a permanent member of G20.^51^ India has stressed disease surveillance, early warning systems, and disaster preparedness in the G20. India has pushed universal health coverage in G20 to ensure everyone can afford basic health services. India’s Quad health diplomacy shows its commitment to regional health issues and Indo-Pacific well-being. The country has actively participated in Quad debates and cooperation to improve health systems, vaccination access, and pandemic preparedness and response.^52^

 India has maintained its commitment to engaging in multilateral global health partnerships by actively collaborating with international organizations such as WHO, its allied programs, and various stakeholders in the global health sector. India was actively engaged in multilateral forums, such as the World Health Assembly, to contribute valuable contributions to global health policies, strategies, and response measures deliberations. India actively engaged in virtual conferences and knowledge-sharing initiatives to disseminate its experiences and best practices in managing the pandemic.

 India actively supported the COVAX initiative, a global collaborative effort to ensure fair and equal distribution of COVID-19 vaccines. COVAX has been created as a global cooperative mechanism that provides an optimistic approach for future pandemics.^53^ Despite its internal vaccine obligations, India exported a significant number of vaccine doses to COVAX, facilitating vaccine distribution to lower- and middle-income countries. This action exemplified India’s dedication to promoting fairness in global health and fostering collaboration among multiple nations. India utilized its technological prowess to assist nations in effectively managing the global pandemic. The country generously disseminated its native COVID-19 diagnostic technology, namely the RT-PCR test kits, to various nations. Besides, India offered online training and capacity-building initiatives to healthcare professionals from collaborating nations to augment their proficiency in the management of COVID-19.

 Fourthly, during the global health emergency caused by COVID-19, India proved to be a trustworthy partner in GHD by supplying vaccines, medications, medical equipment, and training to numerous countries worldwide. India has actively engaged in several international forums and initiatives as part of the worldwide response to the pandemic. India has been working to improve its healthcare system by expanding its testing, immunization, hospital beds, oxygen supply, and research infrastructure. As a provider of vaccines and medical supplies to other countries and as a proponent of an intellectual property right (IPR) waiver for COVID-19 medical products at the World Trade Organization (WTO) platform, India has been an active and reliable stakeholder in GHD during the COVID-19 pandemic and advocated for solidarity, global partnerships, international cooperation to improve access and to ensure equity.^54,55^ Supply of vaccines, pharmaceuticals, and personal protective equipment from India has helped to develop ties between the country and numerous African nations. Together with other developing nations such as South Africa and Brazil, India has been lobbying for a temporary waiver of IPRs on COVID-19 vaccines, medicines, and technologies to make them more accessible and affordable for everyone.^56^ India’s foreign policy towards global health is motivated by its aspiration to be a responsible and caring operator in the global south and strengthen its international standing. India has shown its dedication to the global health agenda and its desire to be a leader in defining the future of global health governance through its participation in GHD during the COVID-19 pandemic.

 Finally, applying its humanitarian approach and implementing humanitarian diplomacy in the field, India has despatched teams of Indian military doctors to countries like Nepal, the Maldives, and Kuwait to help local administrations develop plans to combat the spread of COVID-19. Indian medical staff has conducted online training for their counterparts from other SAARC countries, helping them build their capacities for the management of COVID-19. The Indian response has been much appreciated globally, as is evident from the tweets of various global leaders and messages from the WHO. India’s diplomacy has played a major role in managing the crisis, whether evacuating the distressed people (including foreign nationals) stuck up in Wuhan or facilitating the evacuation of stranded foreign nationals in India. It also started the “Vande Bharat Mission,” a massive airlift operation for its citizens living abroad and repatriated 3 840 000 citizens as of December 2020.^57^

 India’s proactive approach during this crisis, whether in terms of setting the global agenda, engaging in multilateral diplomacy, or highlighting India’s GHD (by supplying drugs and essential medicines, sending teams of doctors, partnering with countries for vaccine development) for global good, or evacuating people in distress from most infected areas will have a long-term impact on how the world views India. Indian response may not have been perfect, but it has certainly underscored that when needed, India can rise to the challenge of managing global crises with a sense of purpose. The current global landscape needs effective leadership, and India’s response to the pandemic within its borders and its engagement with SAARC, GCC, BRICS, and G20 indicates the emergence of a new form of leadership.

 Amidst this context, India is resolute in its determination to enhance regional coordination and cooperation in an effort to halt the spread of the pandemic. It became a major provider of prescription medicines and other crucial medical equipment for COVID-19 treatment. Through its “Vaccine Maitri,” New Delhi has also led global vaccination efforts, supplying hundreds of thousands of Indian-made vaccines to around 71 countries.^58^ India’s approach to GHD is highly informative and may be viewed as an expansion of its broader strategy to rejuvenate and reshape collaborative efforts across many parties. This is especially relevant at a time when multilateral organizations like the WHO are confronting significant challenges to their existence and are in desperate need of reforms. Under this Vaccine Maitri program, as of June 15, 2023, India has supplied a total of 3012.465 doses (in lakhs) of COVID-19 vaccines to 101 countries. These doses include 151.270 doses (in lakhs) in the form of grants, 2340.925 (in lakhs) doses as commercial supply and 520.270 doses (in lakhs) as part of the COVAX arrangement.^59^ The details of vaccine supply by India to some countries are shown below (Table 1)

**Table 1:** Vaccine Supplies to various countries by India

**Country **	**Total supplies **
Netherlands	1136.217
Australia	309.132
Bangladesh	280.828
Myanmar	212.000
Nigeria	98.020
Nepal	94.990
Indonesia	90.150
USA	80.73
Morocco	70.00
Canada	69.859
UK	50.000
Saudi Arabia	45.000
Ethiopia	42.000
Brazil	40.000
Ghana	27.040
Ivory Coast	23.660
Mexico	20.300
Afghanistan	14.680
Sri Lanka	12.640
South Africa	10.000

Note: Supplies so far (In lakhs), as of 15 June 2023). **Source:** https://www.mea.gov.in/vaccine-supply.htm. Accessed July 20, 2023.

## Conclusion

 As a trustworthy and reliable participant in GHD, India has proven its credibility and dependability during the COVID-19 pandemic. The country has actively collaborated with regional and international organizations to address the pandemic by sharing resources and effective coordination with its partners. India has demonstrated its commitment to global solidarity by offering medical supplies, equipment, and expertise to more than 100 countries. During the COVID-19 pandemic, India proved its reliability as a “Global Pharmacy Hub.” The country’s strong pharmaceutical industry has helped developing nations access affordable generic medications. India’s vaccine manufacturing capabilities, specifically the SII, the world’s largest vaccine manufacturer, have played a pivotal role in combating the pandemic.

 India has made significant efforts in health diplomacy at both regional and multilateral levels. The country has actively participated in international organizations, fostered stakeholder collaborations, and significantly contributed to initiatives such as COVAX. India has been a strong proponent of ensuring equitable access to vaccines and treatments, particularly for countries with lower economic resources. It has actively contributed to multilateral forums and knowledge-sharing initiatives by sharing its experiences and best practices in pandemic management.

 India’s rising global leadership can be attributed to its proactive approach, humanitarian diplomacy, and significant contributions to global health initiatives. The actions taken by the entity during the pandemic will have a long-term influence on how it is perceived as a prominent participant in GHD. During this time, India has demonstrated its dedication to global health and proactive approach to leading and fostering collaboration to tackle global challenges. Given these scenarios, it may be contended that India has effectively responded and demonstrated itself as a reliable and trustworthy partner in GHD.

## Acknowledgements

 The researchers thank “Global Health Research and Innovations Canada (GHRIC)” for providing administrative and coordination support for this project.

## Competing Interests

 Vijay Kumar Chattu is one of the editorial board members of *Health Promotion Perspectives*. He is also the Founder and CEO of Global Health Research and Innovations Canada Inc. (GHRIC), Toronto. Other authors declare no competing interests.

## Ethical Approval

 Not applicable.
